# The placental cholinergic system: localization to the cytotrophoblast and modulation of nitric oxide

**DOI:** 10.1186/1478-811X-4-4

**Published:** 2006-05-10

**Authors:** Md Badiul Bhuiyan, Ferid Murad, Michael E Fant

**Affiliations:** 1 Department of Integrative Biology and Pharmacology, University of Texas, Health Science Center at Houston, Houston, Texas, 77030, USA; 2 Department of Pediatrics, University of Texas Health Science Center at Houston, Houston, Texas, 77030, USA

## Abstract

**Background:**

The human placenta, a non-neuronal tissue, contains an active cholinergic system comprised of acetylcholine (ACh), choline acetyltransferase (ChAT), acetylcholinesterase (AChE), and high affinity muscarinic receptors. The cell(s) of origin of placental ACh and its role in trophoblast function has not been defined. These studies were performed to define the cellular location of ACh synthesis (ChAT) in the human placenta and to begin studying its functional role.

**Results:**

Using immunohistochemical techniques, ChAT was observed primarily within the cytotrophoblasts of preterm placentae as well as some mesenchymal elements. Similar intense immunostaining of the cytotrophoblast was observed for endothelium-derived nitric oxide synthase (eNOS) suggesting that ACh may interact with nitric oxide (NO)-dependent signaling pathways. The ability of carbamylcholine (CCh), an ACh analogue, to stimulate a rise in intracellular Ca^++ ^and NO production in trophoblasts was therefore tested using the BeWo^b30 ^choriocarcinoma cell as a model system. First, CCh significantly increased intracellular calcium as assessed by fluorescence microscopy. We then examined the ability of CCh to stimulate NO production by measuring total nitrite/nitrate production in conditioned media using chemiluminescence-based analysis. CCh, alone, had no effect on NO production. However, CCh increased measurable NO approximately 100% in the presence of 10 nM estradiol. This stimulatory effect was inhibited by 1 (micro)M scopolamine suggesting mediation via muscarinic receptors. Estradiol, alone, had no effect on total NO or eNOS protein or mRNA.

**Conclusion:**

These data demonstrate that placental ChAT localizes to the cytotrophoblast and some mesenchymal cells in human placenta. It further suggests that ACh acts via muscarinic receptors on the trophoblast cell membrane to modulate NO in an estrogen-dependent manner.

## Background

The presence of acetylcholine (ACh) in the human placenta, a non-innervated tissue, was first reported in 1933 by Chang and Gaddum [1]. Subsequent studies have documented the presence of all components of the cholinergic system in this tissue [see ref. [2] for a review]. The placenta-derived acetylcholine synthesizing enzyme, choline acetyltransferase (ChAT), was identified and reported by Comline in 1954 and purified to homogeneity by Hersh and Peete in 1977 [3,4]. Fant and Harbison later confirmed the presence of its degradative enzyme, acetylcholinesterase (AChE), and identified the presence of high affinity muscarinic receptors [5,6]. Subsequent studies have confirmed that at least four of the five known muscarinic receptor subtypes and all of the α-subunits of the nicotinic receptor exist in placental tissue [7-10]. However, their temporal and cell-specific expression patterns have not been fully defined.

Harbison and Sastry demonstrated that the placental content of ACh varies with gestational age, reaching a peak at approximately 20–22 weeks gestation and declining toward term [11]. This developmental pattern paralleled the activity of ChAT, suggesting that the placental cholinergic system may be involved in regulating developmental processes relevant to placental growth. The cellular source of placental ACh and its role(s) in placental biology are not known. Initial interests focused on its potential role in regulating placental vascular tone and in regulating amino acid transport. However, those studies have not been conclusive. Carbamylcholine (CCh), an ACh agonist, was shown to stimulate Ca^++ ^uptake in membrane vesicles derived from the microvillous membrane brush border of the human placenta suggesting it may modulate Ca^++^-sensitive signaling events at the plasma membrane [6]. Subsequent studies have also demonstrated the expression of the Ca^++^-dependent, endothelial isoform of nitric oxide synthase (eNOS) in human placenta [12-14]. This isoform has been shown to respond to cholinergic stimulation in other tissues [15,16], suggesting potential signaling interactions may also exist in the placenta. The purpose of this study was to determine the site(s) of ChAT in the human placenta and to examine potential cholinergic/NO signaling interactions.

## Results

### Immunolocalization of placental ChAT and eNOS

**Figure 1:**
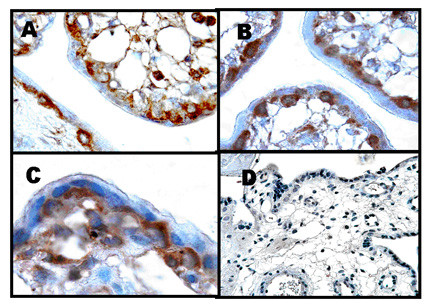
Human placenta obtained at 23 weeks gestation was stained with anti-ChAT antibody as previously described. **Panels A-C: **Positive staining is noted in the cytotrophoblast layer and some cells within the mesenchymal compartment (100×). **Panel D: **Non-immune serum (40×).

Figure 1 represents a placental section obtained at 23 weeks gestation. As shown in **Panels A-C**, the cytotrophoblast stained intensely positive for ChAT. Some placental stromal cells were also positively stained. These cells have not been identified but may represent placental macrophages (Hofbaur cells) since they are known to express ChAT. As seen in **Panel C**, the unstained multinucleated syncytium can be clearly delineated from the underlying, stained cytotrophoblasts. No immunostaining was detected using non-immune serum (**Panel D**). Sections taken from term placentae exhibit immunostaining in the syncytiotrophoblast cell layer, as well, suggesting the mature trophoblast is capable of producing ACh as well (data not shown).

**Figure 2:**
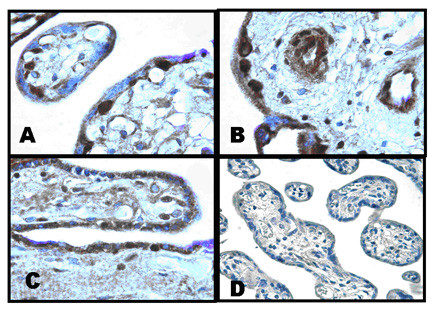
**Panels A – C: **Human placenta obtained at 23 weeks gestation was stained with anti-eNOS antibodies as previously described. Panel A = 100×, Panel B = 100×, Panel C = 40×. **Panel D: **Non-immune serum (40X)

We also noted that the pattern of expression of ChAT was very similar to that observed for the endothelial isoform of nitric oxide synthase, eNOS. In Figure 2, the cytotrophoblast of a 23 week placenta also stained intensely for eNOS. Less intense staining was noted in the syncytium. Some capillary endothelial cells and perivascular stromal cells were also noted to express eNOS immunoreactivity.

### Effect of CCh on Ca^++ ^rise in the BeWo^b30 ^cell

**Figure 3:**
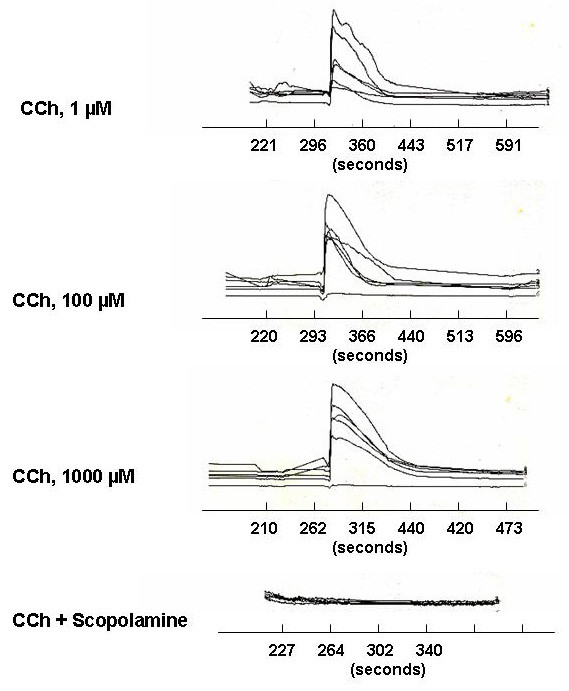
CCh-stimulated Ca^++ ^rise in BeWo^b30 ^choriocarcinoma cells. Intracellular Ca^++ ^was measured in response to various concentrations of CCh, 1 – 1000 (micro)M as previously described. Each line represents a distinct cell. Scopolamine 1(micro)M completely inhibited the rise in CCh-stimulated Ca^++ ^in the cells.

The observed overlap in expression of ChAT and eNOS suggested that the placental cholinergic system could interact with NO-dependent signaling pathways to regulate trophoblast function. To begin to examine this we used the BeWo^b30 ^choriocarcinoma cell line as a model system. This system is an appropriate model because this cell line is derived from human cytotrophoblasts and has been shown to express both eNOS and ChAT [17,18]. Since eNOS activity is sensitive to calcium, we first determined if CCh stimulated Ca^++ ^rise in the BeWo^b30 ^cell. As shown in Figure 3, CCh stimulated a rapid rise of Ca^++^, over a concentration range of 1 (micro)M – 1 mM.

### Effect of CCh on total NO release

**Figure 4:**
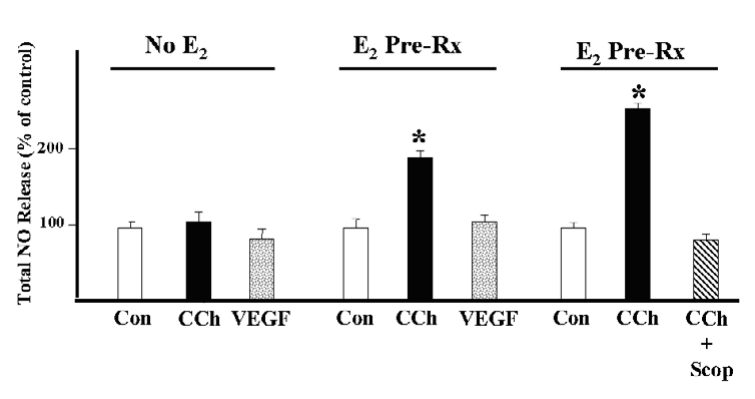
Effect of CCh and VEGF on NO release. BeWo^b30 ^cells were exposed to CCh (10 mM) and VEGF (50 ng/ml) in the presence or absence of 10 nM E2 (estradiol) as previously described. Data represents the mean of triplicate determinations of total media content of NO (nM) from 3 separate experiments. Mean ± S.E.M; * p ≤ .05.

We next sought to determine if CCh stimulated the production of NO in the BeWo^b30 ^cell. To study NOS activity, we determined the release of NO as measured by total nitrite/nitrate content in the conditioned media using a chemiluminescence analyzer. The cells were grown to confluence and placed in serum-free medium 24 for hours. The cells were then treated with 10 mM CCh or 50 ng/ml vascular endothelial cell growth factor (VEGF) for 30 minutes. Based on previously published data [5] using a related choriocarcinoma cell line (JEG-3) we used a concentration of CCh that was necessary to inhibit 80% of [^3^H]-QNB binding to cell surface muscarinic receptors (10 mM) and likely to elicit a measurable effect (*Ki *= 0.13 mM). The *Kd *value for [^3^H]-QNB binding in this cell line is 180–245 pM, consistent with classic muscarinic receptors. Tandem plates were pre-treated with 10 nM estradiol for 16 hours prior to stimulation with CCh or VEGF. Estradiol was dissolved in 100% ethanol at a concentration of 100 (micro)M. This stock solution was diluted to the final contentration of 10 nM in DMEM, 0.1% BSA immediately prior to its addition to the cells. The media was then harvested and analyzed for total nitrite/nitrate content. Ethanol diluted to 0.01% was also added to the cells not containing estradiol to control for potential diluent effects. The cells were washed and re-fed with fresh DMEM, 0.1%BSA prior to the addition of CCh or VEGF. The values were expressed as a percentage of control. As shown in Figure 4, both CCh and VEGF had no effect on NO release in the BeWo^b30 ^cells. However, when the cells were pretreated with 10 nM estradiol, CCh stimulated a 100% increase in NO release in BeWo^b30 ^cells. VEGF, by contrast, continued to have no effect on NO release. Finally, 1 (micro)M scopolamine was able to completely inhibit the effect of CCh in the presence of estradiol suggesting that the effect of CCh was mediated by one or more of the muscarinic receptor subtypes.

### Effect of estradiol on eNOS activity

The mechanism by which estradiol sensitized the BeWo^b30 ^cells to CCh is unknown. We therefore sought to determine if estradiol increased cellular levels of eNOS by assessing eNOS protein levels by immunoblot analysis and eNOS mRNA using semi-quantitative RT-PCR.

**Figure 5:**
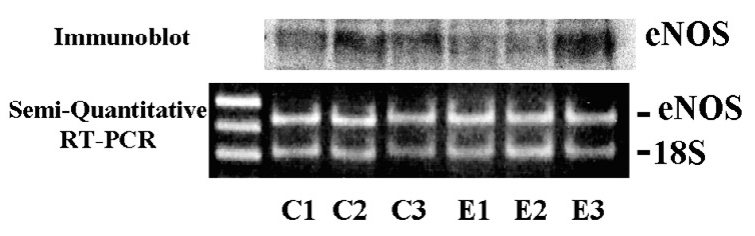
The effect of estradiol on eNOS mRNA and protein expression. BeWo^b30 ^cells were maintained in culture and exposed to estradiol (10 nM) as described. Cellular protein and mRNA were obtained and assessed for eNOS protein by immunoblot analysis and mRNA transcripts by semi-quantitative RT-PCR. Each lane represents a unique sample and is representative of 2 separate experiments. C1, 2, 3 = individual control samples; E1,2,3 = individual E2-treated samples

As shown in Figure 5 estradiol treatment did not increase immunoreactive eNOS or eNOS mRNA. These data suggest that estradiol does not up-regulate total cellular eNOS in the BeWo^b30 ^cell. The possibility that estradiol increases enzymatic activity has not been examined.

## Discussion

We have demonstrated that multiple placental cells express immunoreactive ChAT. Preterm placentae strongly express ChAT in the cytotrophoblast as well as some stromal elements. This is consistent with the report by Sastry and Janson [18] demonstrating the presence of ChAT enzymatic activity in BeWo and JAR choriocarcinoma cell lines. The ability of multiple placental cell types to express ChAT, as indicated by this study, suggests it potentially regulates a variety of cell functions within the placenta. We have previously shown that the placenta expresses muscarinic receptors [5,6]. Subsequent reports by others have suggested that multiple muscarinic receptor subtypes (M1-M4) as well as all subtypes of the nicotinic receptor α-subunit are present in the placenta [7-10]. Each receptor subtype possesses distinct signaling capabilities and thus determines the pharmacologic and biologic specificity of cholinergic agonists. Potential cellular targets of cholinergic stimulation, therefore, are likely to include several cell types influencing cell proliferation, ion flux, secretory processes, cell motility, and cell differentiation. The cell-specific expression of these receptor subtypes and their expression throughout gestation have not been defined but are likely to define important determinants of its cholinergic responsiveness.

Placental ChAT expression overlaps that of eNOS, suggesting that locally produced acetylcholine may stimulate eNOS activity via Ca^++^-dependent mechanisms. The regulation of calcium flux in the trophoblast is critical to fetal and placental development. Molecular systems involved in the cellular uptake and extrusion of calcium have been identified in the BeWo^b30 ^choriocarcinoma cell [19,20]. We have provided evidence that CCh, an acetylcholine analog, can stimulate a rise in cellular Ca^++ ^and NO release via muscarinic receptor-mediated pathways (scopolamine sensitive) in the BeWo^b30 ^cell line. The mechanism(s) by which CCh modulates intracellular Ca^++ ^in this cell line is not known. Interestingly, this stimulatory effect requires the pretreatment with estradiol. The mechanism(s) by which estradiol sensitizes the cell to CCh was not determined. Possibilities include genomic as well as non-genomic mechanisms. Based on these studies, estradiol pretreatment does not appear to regulate eNOS protein levels or gene expression. We have not determined if estradiol regulates functional aspects of eNOS activity or alters its subcellular location that may facilitate modulation of eNOS activity, independent of its expression level. Alternatively, pathways important for NO degradation may be affected, resulting in increased measurable NO.

The role of CCh-modulated NO in the trophoblast is not known. Clearly secreted NO may play roles in maintaining low resistance in the maternal and fetal vascular compartments. Several studies have suggested that NO may play a role in angiogenesis and cell differentiation [21-23]. Additional reports by Sakuragawa and colleagues [24,25] have demonstrated non-neuronal ACh in amniotic epithelial cells, as well, indicating that cholinergic regulatory activity is ubiquitous at the maternal-fetal interface. The physiological significance of ACh-regulatable NO at the placental-maternal interface remains to be established.

## Conclusion

This report demonstrates that ACh modulates the release of NO by cells of trophoblastic lineage in an estrogen-dependent manner. Additionally, they establish that the expression of ChAT overlaps the expression of eNOS in the human placenta suggesting that these signaling interactions are likely to be physiologically relevant at the maternal-fetal interface. Collectively, these findings support the hypothesis that the placental cholinergic system interacts with nitric oxide and estrogen signaling pathways to regulate placental cell growth and/or function.

## Methods

### Source of placental tissue

Human placental tissue was obtained at various gestational ages immediately after delivery in accordance with a protocol approved by the University of Texas-Houston Medical School. The tissue was rinsed and fixed in phosphate-buffered formalin for 16–24 hours and imbedded in paraffin.

### BeWo^b30 ^cell culture

The b30 clone of the BeWo choriocarcinoma cell line was propagated in Dulbecco's Modified Eagles Medium (DMEM) supplemented with 10% fetal bovine serum (FBS) in the presence of 100 units/ml penicillin and 100 (micro)g/ml streptomycin. When the cells were approximately 90% confluent, they were washed in serum free media and placed in Keratinocyte Basal Media (KBM), 0.2% BSA, for 48 hours. Total RNA was then obtained using the RNAeasy kit (Qiagen) per protocol. The RNA was then size fractionated on a 1.5% formaldehyde agarose gel to assess RNA quality. Cellular protein was obtained by lysis of confluent plates of cells using 1 ml of standard RIPA buffer (150 mM NaCl, 10 mM Tris, pH 7.2, 0.1% SDS, 1.0% Triton X-100, 1% Na-Deoxycholate, 5 mM EDTA) in the presence of 1 mM PMSF (phenylmethylsulfonyl fluoride) and 100 uM Na-orthovanadate. The cells were lysed on ice and detached by scraping. The detached cells were then aspirated through a 22 gauge needle 10 times and centrifuged at 10,000 × g for 5 minutes and the pellet discarded. Protein content of the cell lysate was determind using the Bio-Rad Protein Assay ^DC ^per protocol.

### Immunohistochemistry

Immunoreactive ChAT was detected using standard immunohistochemical methods using a monoclonal antibody specific for ChAT (Chemicon) following the Vectastain Elite protocol as previously described [26]. Similarly, eNOS was detected utilizing a monoclonal antibody purchased from BD Biosciences (San Jose, California). Small 0.5 × 0.5 cm. segments were cut and rinsed in ice cold PBS. The tissue was then placed in phosphate buffered formalin and imbedded in paraffin. Sections were cut, deparaffinized and washed × 3 in phosphate buffered saline, pH 7.4 (PBS) followed by 1% H_2_O_2 _in PBS to block endogenous peroxidase activity. The sections were then incubated for 30 minutes at 22°C in normal goat serum (1:50 dilution in 1% bovine serum albumin). The tissue was then rinsed and incubated overnight with a 1:500 dilution of anti-ChAT antibody at 4°C. Finally, the sections were washed and incubated with secondary antibody, biotinylated goat, anti-mouse IgG 1:200 in 1:200 normal goat serum with 1% BSA, for 30 minutes at 22°C. The sections were then washed and incubated with Vectastain (Vector Laboratories, CA) avidin:biotinylated enzyme complex (ABC) 30 minutes followed by 3-3'diaminobenzidine (DAB) for 3 minutes at 22°C per instructions. Finally, the sections were counterstained with hematoxylin. Tandem sections were incubated with 1:500 dilution of non-immune mouse serum to identify non-specific staining.

### Measurement of Ca^++ ^flux

Glass coverslips with cells were incubated for 10 minutes at 37°C with the fluorescent molecule FLUO4-AM, 3 micromolar final concentration (Molecular Probes, Eugene OR) in DMEM buffered with HEPES. Cells were rinsed with DMEM and then transferred to the heating stage. 1.0 ml of DMEM was added to the chamber and placed on the microscope. Measurements of fluorescence intensity of the Ca^++ ^fluoroprobe and sequential image recording of events were made on a Perkin Elmer (Gaithersburg, MD) Concord system incorporating a SpectraMaster multi-wavelength controller. Images were captured by an Olympix AstroCam CCD4100 Fast Scan camera (12 bit; 768 × 576: 1000 frames/sec; 9 micron resolution) every 43 milliseconds. Fluorescence data was analyzed with a Merlin High Performance Ratio Fluorescence Workstation (Olympus America, Melville, NY). Baseline data was taken for 15–20 seconds, then carbachol was added and data acquisition was continued for 1–2 minutes.

### NO quantitation

A chemiluminescence method (Sievers #280 NOA Instruments, Boulder, CO) was used to measure total nitric oxide (NO) as previously described [27]. Briefly, NO released by cells in culture is immediately converted to its oxidative products nitrate and nitrite. Vanadium chloride III, a very strong reducing agent, was used to reduce nitrate and nitrite into NO gas. Sampled gas reacts with ozone to produce activated nitrogen dioxide (NO_2_). NO_2 _reverts to the ground state by emitting electromagnetic radiation that is detected by a photomultiplier tube and generates a computerized digital signal. This signal is expressed quantitatively as NO concentration. A standard curve based on known nitrate concentrations was used to calculate unknowns and the observed values were expressed in nanomolar concentrations.

### Semi-quantitative RT-PCR

eNOS mRNA was measured using relative RT-PCR standardized against 18S RNA using the Quantum RNA Kit (Ambion) per protocol. Primer sequences for human eNOS were as follows:

Forward: 5'-CTGCTGCCCGAGATATCTTC-3'

Reverse: 5'-AAGTAAGTGTGAGAGCCTGGCGCA-3'.

This produced an approximately 421 bp fragment derived from base positions 2158–2579 of the coding sequence. The 18S internal control represented a fragment of 324 bp. Briefly, the linear range for eNOS amplification was determined (25 cycles) and subsequent assays performed under these conditions. 18S primer:competimer ration of 2:8 was found in preliminary experiments to yield optimal results, relative to eNOS abundance. Reverse transcription was carried per protocol at 42°C. PCR was then carried out following the vendors protocol for 25 cycles (94°C × 30 sec, 55°C × 30 sec, 72°C × 30 sec.).

### Immunoblot analysis

BeWo^b30 ^cells were grown in culture. 50 (micro)g aliquots of total BeWo^b30 ^cell lysate was subjected to 4–20% SDS-PAGE under reducing conditions followed by transfer to nitrocellulose membranes. The membranes were then blocked in 5% dried milk and incubated for 2 hours in anti-eNOS antiserum (1:500 dilution) followed by washing and detection using a chemiluminescence detection system (Amersham) per instructions.

### Statistical analyses

The effects of different treatments on measurable NO, and their interactions, were assessed using a 2-way factorial analysis of variance (ANOVA).

## Competing interests

The author(s) declare that they have no competing interests.

## Authors' contributions

**Md. Bhuiyan **performed all of the experiments described under the direct supervision of Drs. Fant and Murad. Dr. Bhuiyan wrote and revised the manuscript with input from Drs. Fant and Murad.

**Dr. Murad **provided expertise and resources used for the measurement of nitric oxide and eNOS. He also was involved with the experimental design, data analysis, and manuscript preparation,

**Dr. Fant **conceived the studies described and supervised Dr. Bhuiyan's laboratory efforts. He played a major role in designing the experiments, data analysis, and manuscript preparation.
